# A systems approach to enable effective team science from the internal research program of the National Center for Advancing Translational Sciences

**DOI:** 10.1017/cts.2021.811

**Published:** 2021-07-12

**Authors:** Amanda L. Vogel, Ann R. Knebel, Jessica M. Faupel-Badger, Lili M. Portilla, Anton Simeonov

**Affiliations:** 1 National Institutes of Health (NIH); National Center for Advancing Translational Sciences (NCATS); Office of Policy, Communications and Education; Education Branch; Bethesda, MD, USA; 2 National Institutes of Health (NIH), National Center for Advancing Translational Sciences (NCATS), Division of Preclinical Innovation (DPI), Office of the Scientific Director, Rockville, MD, USA; 3 National Institutes of Health (NIH), National Center for Advancing Translational Sciences (NCATS), Office of Strategic Alliances (OSA), Rockville, MD, USA

**Keywords:** Translational science, preclinical translational research, team science, collaboration, cross-disciplinary research

## Abstract

The internal research program of the National Center for Advancing Translational Sciences (NCATS) at the National Institutes of Health aims to fundamentally transform the preclinical translational research process to get more treatments to more people more quickly. The program develops and implements innovative scientific and operational approaches that accelerate and enhance translation across many diverse projects. Cross-disciplinary team science is a defining feature of our organization, with scientists at all levels engaged in multiple research teams. Here, we share our systems approach to nurturing cross-disciplinary team science, which leverages organizational policies, structures, and processes. Policies including the organizational mission statement, principles for ethical conduct of research, performance review criteria, and training program objectives and approaches reinforce the value of team science to achieve the program’s scientific goals. Structures including an organizational structure designed around solving translational problems, co-location of employees in a single state-of-the-art scientific facility, and shared-use laboratories, expertise and instrumentation facilitate collaboration. Processes including fluid team assembly, specialized project management, cross-agency partnerships, and decision making based on clear screening criteria and milestones enable effective team assembly and functioning. We share evidence of the impact of these approaches on the science and commercialization of findings and discuss pathways to broad adoption of similar approaches.

## Introduction

The NIH National Center for Advancing Translational Sciences (NCATS) was established to address the pressing need to accelerate and enhance the process of turning observations in the laboratory, clinic, and community into interventions that improve the health of individuals and the public – from diagnostics and therapeutics to medical procedures and behavioral changes. Toward this end, the Center provides national leadership for the field of translational science [1]. The field aims to create and test scientific innovations (e.g., methods, technologies, resources) that enhance the development, testing, and implementation of these interventions. Translational science focuses on increasing scientific efficiency by reducing, removing, or bypassing costly and time-consuming bottlenecks, and enhancing scientific impact via innovation and scalability.

Cross-disciplinary team science – i.e., two or more individuals from different disciplines working interdependently toward a shared scientific goal [2] – is a core strategy of translational science. Evidence supports that this approach can accelerate innovation and breakthroughs and produce more holistic findings with greater relevance to health interventions [2,3]. The NCATS internal research program, housed in the Division of Preclinical Innovation (DPI), leverages cross-disciplinary team science as an essential approach to achieving complex translational goals.

DPI is NCATS’ engine for creating and testing innovative strategies to enhance the preclinical translation process. Its overarching goal is to fundamentally transform therapeutic discovery and development to enhance its efficiency, effectiveness, and impact on human health. The Division’s initiatives span from early discovery through late-stage drug development, to licensing and commercialization, to first-in-human studies.

All DPI research activities are pursued via team science among DPI colleagues, with collaborators at other NIH Institutes and Centers (ICs), and/or with collaborators at external institutions and agencies (e.g., universities, industry). Table 1 shows the distribution of types of DPI collaborations from 2016 through 2020 compared to the average for the internal research programs of four ICs that have budgets of similar size to the NCATS budget. In each year, every DPI scientist was involved in these collaborations. Table 1 also compares the number of each type of collaboration at NCATS to the average number for the four comparator ICs (NIH RePORTER).

**Table 1.** Types of collaborations in internal research programs: comparing NCATS to the average of four other NIH Institutes and Centers with similarly sized budgets.

	2016		2017		2018		2019		2020	
	NCATS	NIH AVG.^*^	NCATS	NIH AVG.^*^	NCATS	NIH AVG.^*^	NCATS	NIH AVG.^*^	NCATS	NIH AVG.^*^
Total projects	175	89.3	182	68.2	184	83	178	84.5	167	87.8
Internal projects	23	18.3	21	13.8	16	17.5	19	19.75	22	22.8
NIH collaborator only	32	9.8	27	7	34	9.5	29	9	23	9.0
External collaborator only	94	28.0	107	21.2	97	24	93	23.5	84	25.5
NIH and external collaborators	26	33.3	27	26.2	37	32	37	32.25	38	30.5

NCATS, National Center for Advancing Translational Sciences; NIH, National Institutes of Health; Avg., average.

* Average across four NIH Institutes and Centers with budgets of similar size to NCATS.

Team science has been central to the DPI culture since the Division was established nearly 10 years ago. The DPI scientific environment was designed with the explicit goal of fostering innovative, dynamic, and outcomes-oriented science via cross-disciplinary and cross-agency collaborations. The planners identified and integrated best practices from pharmaceutical, biotechnology, academic, and government organizations to create a hybrid organizational environment that maximally enables non-hierarchical, project-based, cross-disciplinary team science. Furthermore, the newly formed Division hired experienced scientists with a team science orientation who brought both depth and breadth of experience in preclinical translational team science.

Today, DPI is home to nearly 300 scientists and staff, including trainees, who together have expertise across the preclinical translational space including systems biology; chemical synthesis and optimization; informatics, including machine learning/artificial intelligence; the regulatory requirements for drug development; and public–private partnerships. Scientists at all levels of the organization participate in multiple cross-disciplinary science teams that leverage this diverse in-house expertise, state-of-the-art laboratories, and collaborative relationships across government, industry, academia, and the rare disease community to develop and test novel translational science innovations and achieve scientific breakthroughs.

Tackling translational challenges often begins with projects that focus on specific diseases, disorders, or exposures. DPI scientists develop translational science innovations in these contexts, while also considering how these innovations can be applied more broadly to advance research on a wide range of diseases and conditions. One example is platform technologies that support multiple studies, such as quantitative high-throughput screening technologies, that enable potency assessment of active molecules on a massive scale. Another example is developing methods for de-risking potential drug targets or making them more attractive for commercial investment. Using this approach, DPI has produced notable scientific breakthroughs such as development of a promising drug candidate to treat cancer metastasis and repurposing of FDA approved drugs to identify new therapeutics and novel drug combinations to treat multidrug-resistant bacteria [4,5].

## A Systems Approach to Enable Effective Team Science

DPI’s robust scientific environment is undergirded by an organization-wide system that enables effective cross-disciplinary team science. Our approach facilitates effective team formation and functioning and eliminates multiple common disincentives and challenges to participating in team science (e.g., legacy recognition and reward systems, the added administrative and time burdens involved in project management) [6,7].

**Fig. 1.**
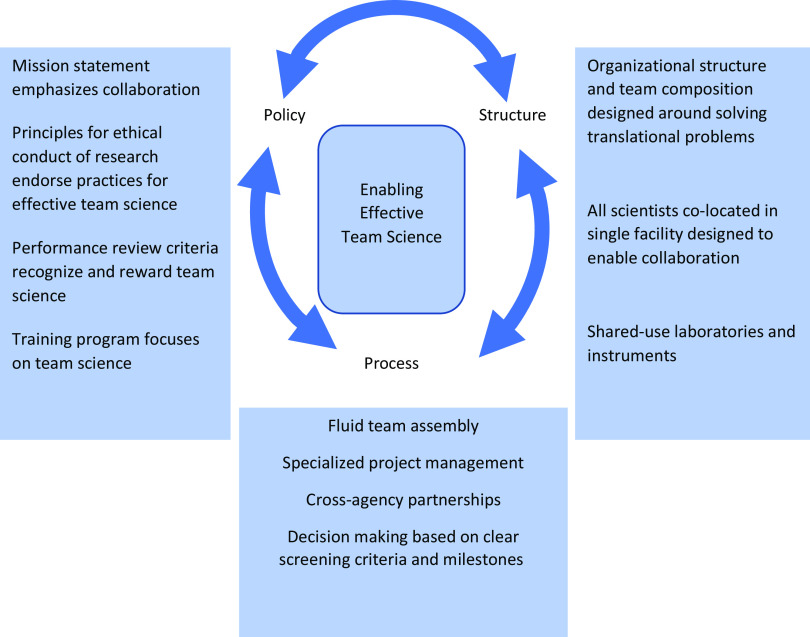
DPI’s systems approach to enable effective team science.

This systems approach leverages organizational ***policies*** that emphasize the importance of team science to achieving the organization’s scientific mission, ***structures*** that facilitate collaboration, and ***processes*** that enable effective and efficient team assembly and functioning (Fig. 1). Ultimately, these factors work together to maximally support effective team science. Here, we describe our approach in detail and share evidence of its impact on the science and commercialization of findings.

### Policies

#### Mission statement and principles for ethical conduct of research link team science to achieving scientific goals

The mission of DPI is to “transform therapeutic discovery approaches and tools; advance the art of collaboration; and catalyze the biomedical community to deliver the most effective therapies to treat human disease.” This mission statement provides a strategic vision for DPI that is centered around our philosophy of collaboration and sets the course for team science to achieve our scientific goals.

Acculturation to the centrality of team science to DPI’s mission begins with a prospective employee’s initial interview. Expectations around engagement in cross-disciplinary science are articulated and candidates are screened for a team science orientation with values- and behavior-based interview questions, as outlined in the Team Science and Collaboration Field Guide [8]. Preference is given to individuals who have prior experience working in a team science environment. The centrality of team science to the DPI culture is reinforced through a required team science performance element in the annual performance evaluations for all federal staff. The element demonstrates the value DPI places on team-based collaboration; conflict resolution; and for supervisors, establishing clear expectations for collaboration and shared credit.

The DPI principles for ethical conduct of research define behaviors that are foundational both to the Division’s culture of team science and to ethical scientific conduct. Specifically, the principles highlight the importance of role clarity, effective communication, skillful management of conflicts, and credit for individual contributions to team science to maintain effective collaborations, build trust, and ultimately ensure research integrity.

DPI reinforces the principles for ethical conduct of research through annual ethics case discussions. Cases describe ethical conflict scenarios in the research environment and facilitators lead participants in discussing potential approaches to address them. DPI facilitators adapt cases provided by NIH for relevance to our team science environment.

#### Performance review criteria recognize and reward team science

Recognition and reward policies are a key influence on scientists’ level of engagement in cross-disciplinary team science [6,9]. Multiple advisory bodies have issued reports that call for widespread revision of promotion and tenure policies to recognize and reward team science [10,11].

Aligned with these recommendations, DPI has no Principal Investigators (PIs) and no tenure, and therefore lacks a PI-driven promotion and tenure approach. Rather, all DPI scientific programs are reviewed every 4 years by a panel of external reviewers. The review has two foci: overall programmatic accomplishments (ad hoc scientific review) and the contributions of individual scientists to the research conducted by the program (quadrennial or Quad review). Reviewers are drawn from academia, industry, and other federal agencies – there are no internal reviewers – and are selected based on their scientific expertise relevant to the programs being reviewed. Reviewers are oriented to the DPI structure and team science environment as well as DPI’s performance review criteria. They apply their scientific expertise to evaluate programs and individual contributions and accomplishments using the performance review criteria.

**Fig. 2.**
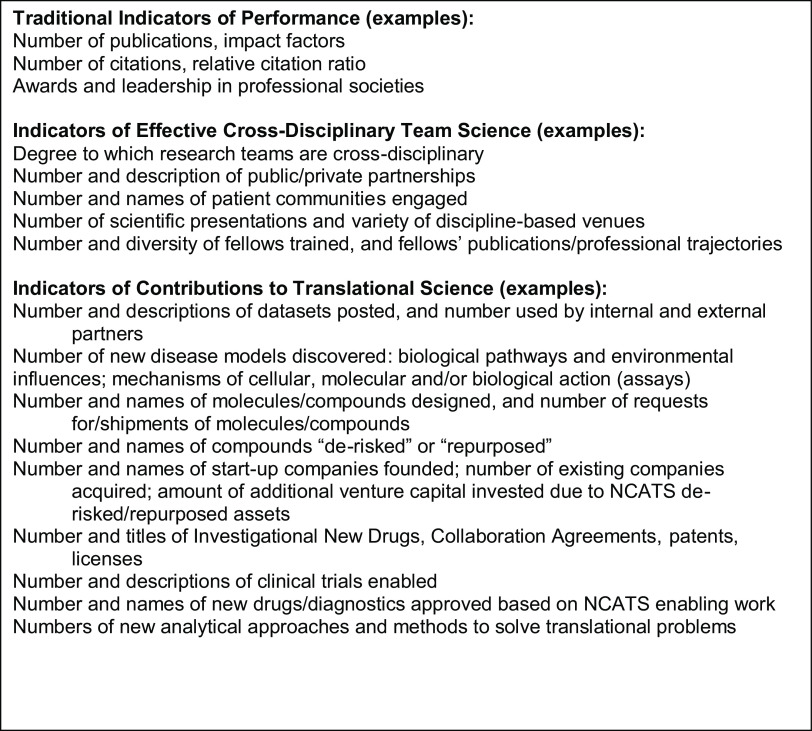
DPI staff/senior scientist performance review criteria examples.

DPI’s performance review criteria include traditional indicators of performance (e.g., indicators of productivity, impact, and scholarly reputation), novel indicators of effective cross-disciplinary team science, and indicators of contributions to translation (Fig. 2). The team science-specific indicators reflect the interdependence that characterizes high functioning science teams and the disciplinary breadth that is a key benefit of effective cross-disciplinary team science [12]. Overall, DPI’s performance review criteria free DPI scientists to work on a breadth of translational problems, unbounded by field or discipline, and to do so in teams with diverse expertise.

A key aspect of the DPI Quad review is understanding the individual contributions of each participant on the science team. DPI scientists are encouraged to use the CRediT taxonomy to articulate their individual contributions [13]. The taxonomy identifies 14 typical contributions to scientific scholarly output, including conceptualization, data curation, project administration, resources, and others. Reviewers critique the contributions of individual scientists and those who do not meet expectations are given a performance improvement plan or their appointments may not be renewed.

#### Training program includes a focus on team science

Developing the translational science workforce is one of the four strategic goals of NCATS [14]. Intramurally, NCATS pursues this goal through the DPI training program, which is aligned with the overall mission of the NIH Intramural Research Program to “train the next generation of biomedical and behavioral researchers.” The Division’s training program includes a range of opportunities from summer internships to 1–5-year fellowships for post-baccalaureate, graduate student, and postdoctoral fellows [15].

The DPI training program emphasizes development of transferrable translational science skills and knowledge, distinguishing it from traditional research training opportunities that build expertise in a focused area of science. There is a strong focus on developing skills and knowledge for cross-disciplinary team-based translational science.

Trainees receive both experiential training and educational opportunities [15]. Trainees are full participants in multiple team-based projects and receive multi-mentoring from scientists with varied disciplinary backgrounds and expertise – both of which are tested approaches to team science training [16,17]. Fellows conduct research and participate in team meetings and strategy discussions, providing rich opportunities to learn team science skills first-hand. In addition, they are encouraged to produce first-author publications in a focused area of research and submit project proposals to the Opportunities Committee, described below. If their proposals are funded, they can lead their own pilot project teams.

In addition, all trainees participate as speakers and audience members in a seminar series that includes presentations from all programs and research disciplines represented in DPI, providing additional training for cross-disciplinary science. They also participate in courses built around case studies of effective DPI team science initiatives, which teach scientific and operational principles of translational science [15,18].

### Structures

#### Organizational structure and team composition designed around solving translational problems

The DPI organization was designed to facilitate the process of solving translational problems. Team composition approaches vary by organizational unit (Branch or Core) to best accomplish that unit’s unique work. Overall, the DPI organization is composed of three Branches and three Core Functions. In the *Early Translation Branch,* there are cross-disciplinary teams of biologists, chemists, and informaticians. In the late-stage *Therapeutic Development Branch (TDB),* teams are composed around disciplinary expertise relevant to the later stages of therapeutic development: biology, medicinal chemistry, pharmacokinetics, toxicology, formulation, and manufacturing, as well as project management. In the *Chemical Genomics Branch*, teams are structured around programs that have been on-boarded to address key translational problems such as functional genomics (a trans-NIH collaborative core facility), stem cell translation (established with funding from the NIH Common Fund), and testing toxic effects of chemicals (Toxicology for the 21st Century, an interagency partnership with the FDA, EPA, and the National Institute of Environmental Health Sciences (NIEHS)).

DPI’s three Core Functions are *Informatics, Analytical Chemistry, and Research Services* (which includes automation and compound management). The Core Functions provide services across all the DPI programs and simultaneously pursue their own programs of research. For example, the Analytical Chemistry Core provides analytical support to the teams in the Branches and has a research program in high-throughput proteomics. This dual role was designed to maximize the value of the Cores to translational problem solving.

The Branches and Core Facilities are led by Directors who provide the administrative infrastructure (i.e., budget, human resources, space) to enable translational science. They do not promote their own programs of research but work with team leads within and across the Branches and Cores to address translational problems. This model, which is distinct from the hierarchical model found in traditional PI-driven labs, encourages DPI scientists to collaborate with one another on multiple projects within and across Branches and Cores to effectively address translational science challenges.

#### All DPI scientists and trainees are co-located in a single facility designed to enable collaboration

Collaboration can be enhanced or undermined by the design of the physical workplace. Shared physical spaces, path overlap (overlap in functional walking space), and physical proximity of office spaces all increase the likelihood of forming new collaborations [3,19].

The NCATS internal research program is housed in a single scientific facility that is designed to enable cross-contact among Branches and Cores. The DPI research building offers 80,000 square feet of laboratory, office, and meeting spaces. Offices for many of the DPI leaders from across different organizational units are located on the third floor of one wing of the building. Individual offices are on the periphery and central areas consist of cubicles and shared spaces. This generates path overlap and opportunities to congregate informally that enhance impromptu contact across components and levels of the organization. For example, the Director of the Analytical Chemistry Core sits across from the open area where the Informatics Core staff are located, and adjacent to the offices of the Early Translational Branch leadership. In addition, many offices for scientific staff are located adjacent to the labs on the first and second floors, again creating opportunities for unplanned interactions that can stimulate new collaborations and enhance communication within and across teams. Finally, the NCATS Office of Strategic Alliances (OSA), described below, is also co-located with DPI to increase interactions among OSA and DPI staff.

#### Shared-use laboratories and instruments

Shared resources within an organization can facilitate creation of new collaborations [3]. The DPI state-of-the-art laboratories house a broad array of capabilities under one roof. A few examples include high-throughput screening technology, RNAi screening, well-designed chemical libraries, 3-D tissue bioprinting, stem cell technologies, drug formulation, and medicinal chemistry. Many of the laboratories are shared-use spaces. For example, chemistry labs are shared among chemists across multiple teams and branches, and the same is true of biology labs. Instrumentation is likewise shared. For example, the Stem Cell Lab has optical microscopes that are used by many other teams. DPI encourages shared instrumentation through purchasing decisions. During budget planning for equipment purchases, input is sought from all scientific leads. Emphasis is placed on purchases that can be leveraged for multiple projects and the costs are shared across teams. This approach is dramatically different from the typical approach where a PI determines what equipment to purchase in support of his/her program of research.

### Processes

#### Fluid team assembly

While individual DPI scientists and trainees have organizational “homes” within Branches or Cores, and are members of workgroups and teams within these organizational units, they are encouraged and supported to form additional teams or collaborations with members drawn from across Branches and Cores for particular scientific projects or to address scientific challenges that emerge in their work. Individuals routinely participate in multiple cross-disciplinary teams composed of members from across Branches and Cores. As federal employees, DPI scientists’ salaries are paid according to their hiring mechanisms; they do not seek funding to cover their salaries. Funds are not needed to support collaborations because they are considered a core part of the scientists’ jobs.

To stimulate and support assembly of new cross-cutting teams within the Division, DPI established an Opportunities Committee that in 2019 began funding 1–2-year exploratory pilot projects. The pilot award requires the proposed project team to leverage expertise from across DPI Branches and Cores and to pursue a translational science innovation. Awarded teams may also include external collaborators and may be led by DPI staff or fellows. If the pilot projects are successful, they can be incorporated into the Branch or Core’s portfolio of activities.

#### Specialized project management

Skillful leadership and management are critical to effective team functioning and become increasingly important with growing team complexity, including team size and the diversity of team members’ disciplines and agencies [7,8]. In response to this need, there has been a national trend toward development of career tracks in leadership, management, and administration of large, complex team science initiatives [20,21].

DPI uses a project team matrix model, common to the pharmaceutical sector, to flexibly integrate functional disciplines (e.g., biology, chemistry, informatics, engineering, and robotics) in cross-Branch and Core project teams. These project teams are supported and managed by staff members with specialized roles in project management.

Project management in the Division varies based on the needs of each project, operating along a continuum from purely administrative and managerial duties to scientific co-leadership. In the former category, Project Analysts are scientists who have bachelor’s or master’s degrees and focus on team communication and coordination. They schedule meetings, take and organize meeting notes, facilitate communication among team members, and create tools for tracking project activities and organizing information. In the latter category, Project Managers are scientists who have PhDs and offer high-level scientific co-leadership and management alongside multiple scientific co-leads for the team. Project Managers work on late-stage translational projects, which are the most complex in terms of duration, scientific components, and collaborators, and therefore require the greatest investments in leadership and management.

Scientific training as well as leadership, managerial, and administrative skills are all essential for success in the Project Manager role. The career path begins with scientific expertise; it may include Project Management Professional certification, but that is not required [22]. The Project Manager ensures that everyone on the team understands his or her role in achieving the shared goals. In addition, the Project Manager enables communication, helps to resolve disagreements, and promotes coordinated efforts to achieve milestones. The experience of DPI’s scientific teams is that effective Project Managers increase teams’ efficiency and improve scientific outcomes.

#### Cross-agency partnerships

Strategic collaborations with external agencies are essential to achieving DPI’s goals. DPI relies on these collaborations to harness expertise and technology that complements in-house resources. Examples include collaborators with disease-specific expertise and Biosafety Level 3 capabilities. Collaborators are located at other NIH ICs, academic institutions, the private sector, and nonprofit organizations such as disease foundations and patient advocacy groups. DPI scientists generate novel intellectual property jointly with their collaborators, reflected in the DPI patent portfolio, which is approximately 80% co-owned with academic and for-profit partners.

The NCATS OSA facilitates effective collaborations with these partners to ensure smooth and efficient transitions from the preclinical work of DPI to the next stages on the translational spectrum, including clinical trials and commercialization and licensing. Table 1 shows that DPI has averaged 177 collaborations per year over the past 5 years. OSA specializes in both (1) alliance management and relationship building to lay the foundations for effective partnerships and (2) establishing legal structures and frameworks to successfully form, maintain, and evolve partnerships with external organizations. These legal structures include different types of agreements (e.g., confidential disclosure, research collaboration, research and development, material transfer, etc.) and address all aspects surrounding invention reporting, patenting, and licensing.

OSA engages with DPI scientists early in discussions of DPI projects to understand the nature of the collaboration(s) and provide consultation on the most effective and efficient approaches to implementing the strategic alliance or collaboration. OSA’s co-location with DPI helps its staff stay abreast of the scientific initiatives and programs, which is key to addressing the unique nature of each DPI scientific collaboration. It is critical that OSA have context on the overall strategies and prioritization of a scientific project such as understanding how a collaborator is going to handle data, new intellectual property, and jointly developed research materials, as this leads to better agreement drafting.

OSA has developed numerous template agreements for DPI initiatives that save time and resources on both sides [23]. In order to ensure uptake of these templates, OSA frequently seeks feedback from external users prior to launching agreements for new initiatives. This input helps OSA develop agreements that require minimal modifications, thus leading to quick and efficient execution so the science can get started.

OSA also educates DPI scientists on strategies for working with businesses and industry to translate their novel scientific findings into partnerships that can lead to commercialization of their translational discoveries. OSA recently launched a training program based on the National Science Foundation’s Innovation Corps (I-Corps) Program. Conducted in collaboration with the National Cancer Institute, the Advancing Innovations through Mentorship (AIM) program intends to advance translational discoveries that arise from DPI by empowering NCATS investigators to evaluate their technologies in the context of the commercial and healthcare landscapes. The first AIM pilot cohort consisted of seven teams each with three to four members. Participants found the training valuable (95%) and reported that it would change their research approaches (75%). While the AIM program is new, to develop AIM, OSA leveraged the curriculum of the very successful NIH Small Business Innovation Research (SBIR) I-Corps program geared toward NIH funded SBIR grantees and contractors [24].

#### Decision making based on clear screening criteria and milestones for go/no go decisions

DPI teams have implemented well-defined criteria and processes for decisions regarding uptake of new projects to ensure the projects fit with the strategic vision of DPI and are ones in which DPI can make innovative scientific contributions. This enables internal and external collaborators to establish a shared understanding of the rationale for project uptake decisions.

For example, the Functional Genomics Lab requires that all potential collaborators have a well-defined assay before a project is taken on. If an assay is not available, DPI scientists work with the potential collaborator to develop the assay. Another example is from the NIH Helping End Addiction Long Term (HEAL) initiative, in which NCATS is a participant. The first milestone in any potential collaboration through HEAL is a “proof of concept” analysis, meaning that DPI must be able to replicate the preliminary findings of the collaborator. In addition to these uptake criteria, there are also milestones for project progression that lead to go/no-go decisions. By clearly defining project uptake and continuation criteriaDPI is able to clearly communicate both internally and with external collaborators regarding the rationale behind decisions, reach consensus on these decisions, and redirect resources efficiently. Overall, this approach helps ensure that DPI uses its resources to maximal advantage to advance translational science.

## Discussion

DPI’s systems approach to nurturing team science reflects the NCATS culture of innovation, drawing upon effective practices gleaned from government, academia, and the pharmaceutical industry to create a tailor-made approach that is ideally suited to DPI’s environment and scientific goals. For example, some of DPI’s performance review criteria and the role of the Project Manager reflect practices in the pharmaceutical industry. The cross-Branch and -Core teams that conduct DPI projects reflect the organizing principle of cross-departmental research centers in academic institutions. The OSA fulfills the role of a government Technology Transfer Office and adds to this a unique approach to building and maintaining partnerships.

The approach DPI has developed is unique in its comprehensiveness. Interacting interventions at the levels of policy, structure, and process reinforce one another to remove barriers to team science and facilitate success. This produces an environment with remarkable scientific and operational freedom. DPI scientists are free from the strictures of traditional review criteria, turf wars, and claims to property; supported by policies that recognize the value of cross-disciplinary collaboration; enabled with support for project management and cross-agency partnerships; and housed in a facility with state-of-the-art shared resources. The result is that DPI scientists are engaged in a remarkably diverse range of scientific activities, in partnership with a range of collaborators with varied expertise, and are pursuing ambitious research that solves longstanding and challenging problems in preclinical translation.

Consequently, DPI science is extraordinarily creative. DPI scientists pursue leads that are not limited to a defined disease or program area. They can invest in long-term projects and participate in multiple ambitious initiatives that could not be conducted in a more traditional environment. Further, they have the flexibility to pivot quickly to respond to emerging scientific needs. The resulting scientific output has the potential to dramatically advance therapeutic discovery and development in service to society.

The impact is evidenced in DPI’s scientific productivity, scope, and influence. For example, from 2016 through 2020, DPI scientists coauthored more than 400 journal articles on a broad range of topics, many of which were in high impact journals. NIH does not prescribe a specific number of publications as a benchmark, instead focusing on publication quality, rigor, innovativeness, and impact. A few recent examples illustrate these features in DPI’s publications: a serosurvey of COVID-19 [25]; molecular targets and pathways for organ level toxicity [26]; computational methods in metabolomics [27]; targeting cancer mutations with kinase inhibitors [28]; and therapeutic candidates for the Zika virus [29]. These publications [25–29] demonstrate the quality and public health impact of the research. Table 2 links the major findings of these studies to the performance metrics in Fig. 2, providing a deeper analysis of the scientific contributions of the studies represented in this sample of DPI manuscripts.

**Table 2.** Publication impact and link to review criteria.

Publication topic	Journal	Major finding	Review criteria
Serosurvey of COVID-19	Journal of Infectious Diseases	Within 6 months of identification of SARS-CoV-2, analyzed the serologic reactivity of two variants and determined there was a cross-responsive humoral immunity	New disease model: mechanism of cellular, molecular and/or biological action
Molecular targets and pathways for organ level toxicity	Chemical Research in Toxicology	Deduced molecular targets and biological pathways for chemically induced organ level toxicity: heart, developmental, liver, kidney, reproductive, etc.	New disease model: biological pathway and environmental influence
Computational methods in metabolomics	Metabolites	Describes the common types of analyses performed in multi-omics studies and the analytical methods that can be used for each type of analysis. Describes the application of these methods to clinical and basic research	Explicating novel analytical approaches and methods to solve translational problems
Targeting lung cancer mutations with kinase inhibitors	The Journal of Clinical Investigation	Medicinal chemistry study of a compound with drug-like qualities that inhibits drug resistant FLT3 as a potential treatment of acute myeloid leukemia	Molecules/compounds designed
Therapeutic candidates for the Zika virus	Proceedings of the National Academy of Sciences of the USA	Using high-throughput screening, identified inhibitors of Zika virus infection that have the potential to be used as prophylactics for the treatment of neurological complications of Zika virus infection	Compounds “de-risked” or “repurposed”

Benchmarks in commercialization also reflect the impact of the DPI team science environment. The TDB engages with external stakeholders to help move promising candidate compounds toward commercialization. TDB has successfully de-risked the preclinical development of a diverse portfolio of novel therapeutic candidates, enabling TDB’s collaborators to successfully file 32 investigational new drug (IND) applications that were cleared by both the FDA and Health Canada since 2011. These INDs cover a broad range of therapeutic areas (Table 3).

**Table 3.** Therapeutic areas for filed investigational new drug (IND) applications since 2011.

Therapeutic area	Number
Nervous system	6
Hematology	4
Musculoskeletal	4
Cardiovascular	3
Lysosomal storage	3
Ophthalmology	3
Metabolic	2
Pulmonary	2
Infectious	1
Ocular	1
Oncology	1
Pain	1
Radiation countermeasure	1
Total	32

The US Patent Trademark Office issued 22 US patents developed by DPI staff between October 2018 and July 2020. Many of these patents were developed through DPI collaborative relationships with partners in government, industry, academia, and patient and rare diseases communities. Twenty-seven DPI staff are listed as co-inventors. Overall, from 2008 through 2020, NCATS filed 197 invention reports, including 107 that were joint inventions with collaborators outside of NIH, 39 that were joint inventions with scientists at other NIH ICs, and 41 that were solely developed by DPI scientists [30]. Compared to other similarly sized ICs, DPI had significantly more new patent filings and granted US and international patents between 2017 and 2020. On average, 25 US and international patents are granted per year naming DPI inventors. As for licenses, NCATS executes an average of eight per year resulting from DPI collaborations and partnerships.

In addition, DPI’s approach produces a nimbleness that is unique in both government and academia. DPI’s ability to quickly pivot to address new scientific program areas has been demonstrated numerous times. A few examples follow. In response to the Deep-Water Horizon oil spill, the DPI Toxicology for the 21st Century program quickly evaluated whether the large volumes of dispersants being used to mitigate the spill would have negative effects on key hormonal processes in humans [31]. During the Zika virus outbreak, DPI scientists were able to identify in record time previously approved drugs that could be repurposed to treat the disease [32]. In response to the opioid epidemic, four DPI teams pivoted to address the crisis. For example, the stem cell laboratory developed a new cell differentiation protocol for nociceptors that is highly efficient and scalable, producing billions of functional human nociceptors and opening opportunities for the study of non-addictive pain treatments [33]. In response to the ongoing COVID-19 pandemic, a new biological activity-based modeling (BABM) paradigm was developed to accelerate identification of new chemical classes for rapid development of therapies. This approach builds on the hypothesis that compounds with similar activity patterns tend to share similar targets or mechanisms of action [34].

Finally, the benefits of the DPI scientific environment have enabled DPI to retain many of the remarkably talented scientists who joined the Division when it was founded 10 years ago, even though they could garner higher salaries in the private sector. Recruitment of top talent to government positions can be difficult because of salary limitations, particularly in highly compensated fields such as engineering and informatics. The DPI environment in and of itself helps to recruit and retain top talent because there is less emphasis on individual distinction and more emphasis on the mission of developing translational science methods and technologies to bring more treatments to more patients more quickly.

## Conclusions

The goal of this paper is to share with other organizations DPI’s approach to producing an organization-wide environment that enables effective team science in order to assist in their own efforts to facilitate cross-disciplinary team science to advance translation. Other institutions, particularly among the CTSA hubs, are likewise developing and testing innovations to advance team science in the translational space. Different approaches are likely to be effective in government, private industry, and academia. A number of overarching observations nonetheless apply across these settings. Broader adoption of team science interventions will require the commitment of organizational leadership; the implementation of systems-level approaches that comprehensively address policies, structures, and processes; and broad recognition by the scientific community that meaningful advances in translation can be achieved through the mobilization of cross-disciplinary science teams.
